# Clinical implications of micro lymph node metastasis for patients with gastric cancer

**DOI:** 10.1186/s12885-023-11023-w

**Published:** 2023-06-12

**Authors:** Yuan Tian, Yue Pang, Pei-Gang Yang, Hong-Hai Guo, Yang Liu, Ze Zhang, Ping-An Ding, Tao Zheng, Yong Li, Li-Qiao Fan, Zhi-Dong Zhang, Dong Wang, Xue-Feng Zhao, Bi-Bo Tan, Yu Liu, Qun Zhao

**Affiliations:** Third Surgery Department, the Fourth Hospital of Hebei Medical University, No.12, Jian-Kang Road, Shijiazhuang, 050011 Hebei Province China

**Keywords:** Gastric carcinoma, Gastrectomy, Micro lymph node metastasis, Lymphadenectomy, Prognosis

## Abstract

**Background:**

Lymph node size is considered as a criterion for possible lymph node metastasis in imageology. Micro lymph nodes are easily overlooked by surgeons and pathologists. This study investigated the influencing factors and prognosis of micro lymph node metastasis in gastric cancer.

**Methods:**

191 eligible gastric cancer patients who underwent D2 lymphadenectomy from June 2016 to June 2017 in the Third Surgery Department at the Fourth Hospital of Hebei Medical University were retrospectively analyzed. Specimens were resected en bloc and the postoperative retrieval of micro lymph nodes was carried out by the operating surgeon for each lymph node station. Micro lymph nodes were submitted for pathological examination separately. According to the results of pathological results, patients were divided into the “micro-LNM (micro lymph node metastasis)” group (N = 85) and the “non micro-LNM” group (N = 106).

**Results:**

The total number of lymph nodes retrieved was 10,954, of which 2998 (27.37%) were micro lymph nodes. A total of 85 (44.50%) gastric cancer patients had been proven to have micro lymph node metastasis. The mean number of micro lymph nodes retrieved was 15.7. The rate of micro lymph node metastasis was 8.1% (242/2998). Undifferentiated carcinoma (90.6% vs. 56.6%, *P* = 0.034) and more advanced Pathological N category (*P* < 0.001) were significantly related to micro lymph node metastasis. The patients with micro lymph node metastasis had a poor prognosis (HR for OS of 2.199, 95% CI = 1.335–3.622, *P* = 0.002). For the stage III patients, micro lymph node metastasis was associated with shorter 5-year OS (15.6% vs. 43.6%, *P* = 0.0004).

**Conclusions:**

Micro lymph node metastasis is an independent risk factor for poor prognosis in gastric cancer patients. Micro lymph node metastasis appears to be a supplement to N category in order to obtain more accurate pathological staging.

## Background

Approximately half of gastric cancer (GC) patients have lymph node metastasis (LNM) at the time of initial diagnosis or surgical resection, and the prognosis is poor [1]. LNM is one of the most important factors related to the prognosis of gastric carcinoma [2]. Typically, cancer cells invade the lymph nodes (LNs) and proliferate within the lymphatic system, causing corresponding LNs enlargement. In some studies, metastatic LNs were defined as those with a diameter > 10 mm along the short axis or a short/long axis diameter ratio of 0.75 on computed tomography (CT) [3, 4]. The diagnosis of N category is only based on the number of positive nodes according to the 8th Edition of the AJCC Cancer TNM Classification [5]. Thus, the diagnosis of bulky LNs is emphasized in the clinic, and it is easy to ignore the small size of LNs. It is often considered that the retrieval of micro lymph nodes (micro-LNs) increases the clinical workload, and micro-LNs have almost no possibility of metastasis.

The Fourth Hospital of Hebei Medical University Gastric Surgery Study group has summarized a series of practical LN sorting strategies for better application of lymph tracers to achieve standardized LN dissection [6, 7]. Thus, our previous study has proposed the concept of micro-LNs. The micro-LN is defined as the LN with a maximum diameter less than 2 mm [8]. Micro lymph node metastasis (Micro-LNM) refers to the invaded micro-LN by tumor cells that is related to the lymph nodal diameter, but not to the tumor size. However, lymph node micrometastasis (LNMM) is considered as LNM with a tumor size of ≤ 2000 μm in metastatic LNs [9]. Note that in our study micro-LNM and LNMM are two separate concepts. To the best of our knowledge, no studies have proven the necessity of the retrieval of micro-LNs and the influence of micro-LNM. The purpose of this retrospective study was to explore the clinicopathological characteristics of micro-LNM and the prognosis of GC patients with micro-LNM. We consider the micro-LNM to be as important as metastases in larger LNs. Micro-LNM has clinical significance and should be paid attention to by surgeons.

## Methods

### Study population

**Fig. 1:**
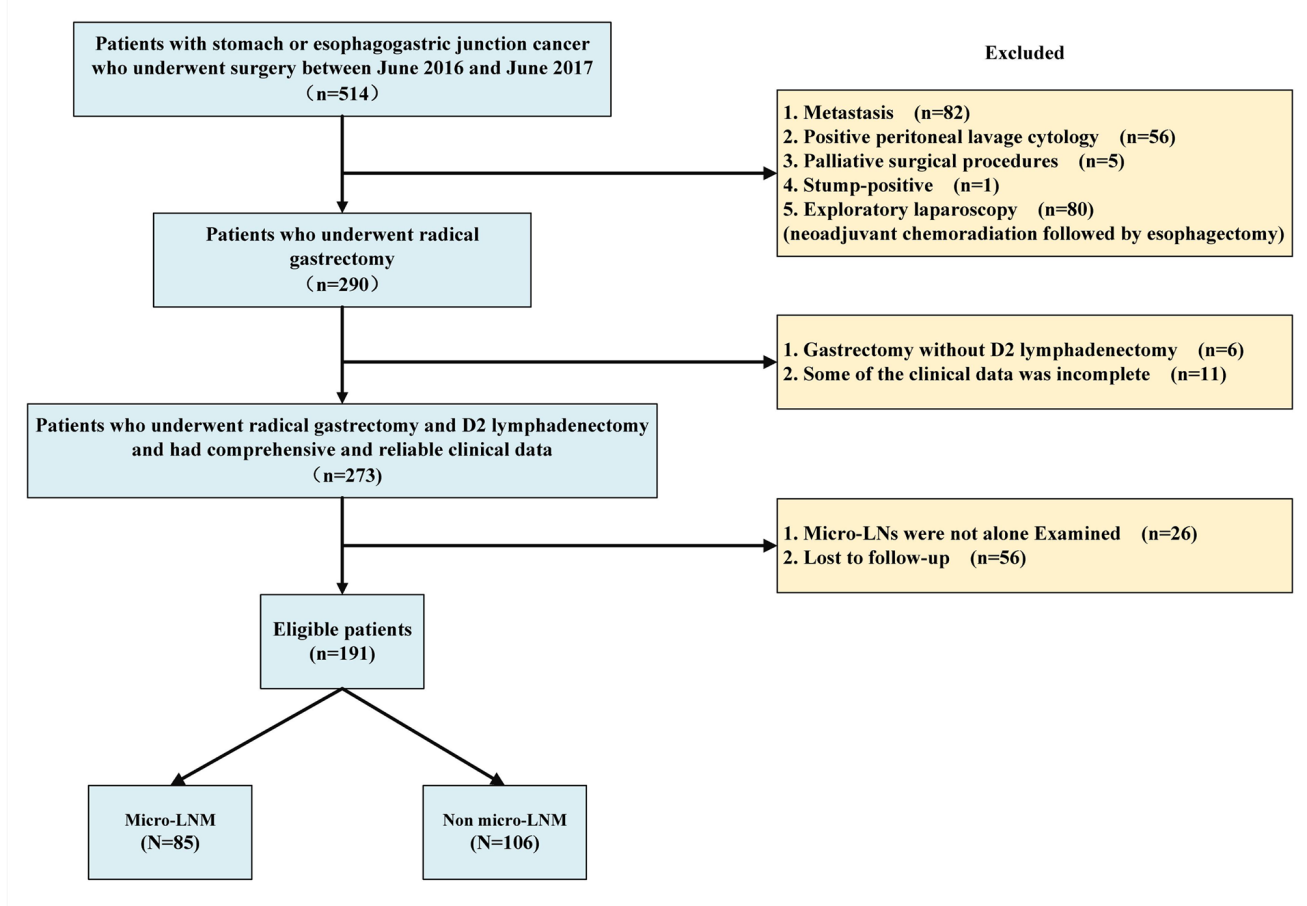
Flow chart showing the patient selection process

The clinical and pathological data of 191 patients undergone radical gastrectomy and D2 lymphadenectomy from June 2016 to June 2017 at the Fourth Hospital of Hebei Medical University in China were retrospectively analyzed. The inclusion criteria were as follows: (1) histologically confirmed adenocarcinoma of the stomach or esophagogastric junction and (2) gastrectomy with standardized D2 lymphadenectomy. The exclusion criteria were as follows: (1) laparoscopic exploration showing positive peritoneal metastasis or peritoneal free cytology, (2) received neoadjuvant chemotherapy or radiotherapy, (3) required palliative surgical resection, and (4) combined resection of other organs. The patient selection is shown in Fig. 1. All patients were informed and signed written informed consent. This retrospective study was approved by the Ethics Committee of the Fourth Hospital of Hebei Medical University.

**Fig. 2:**
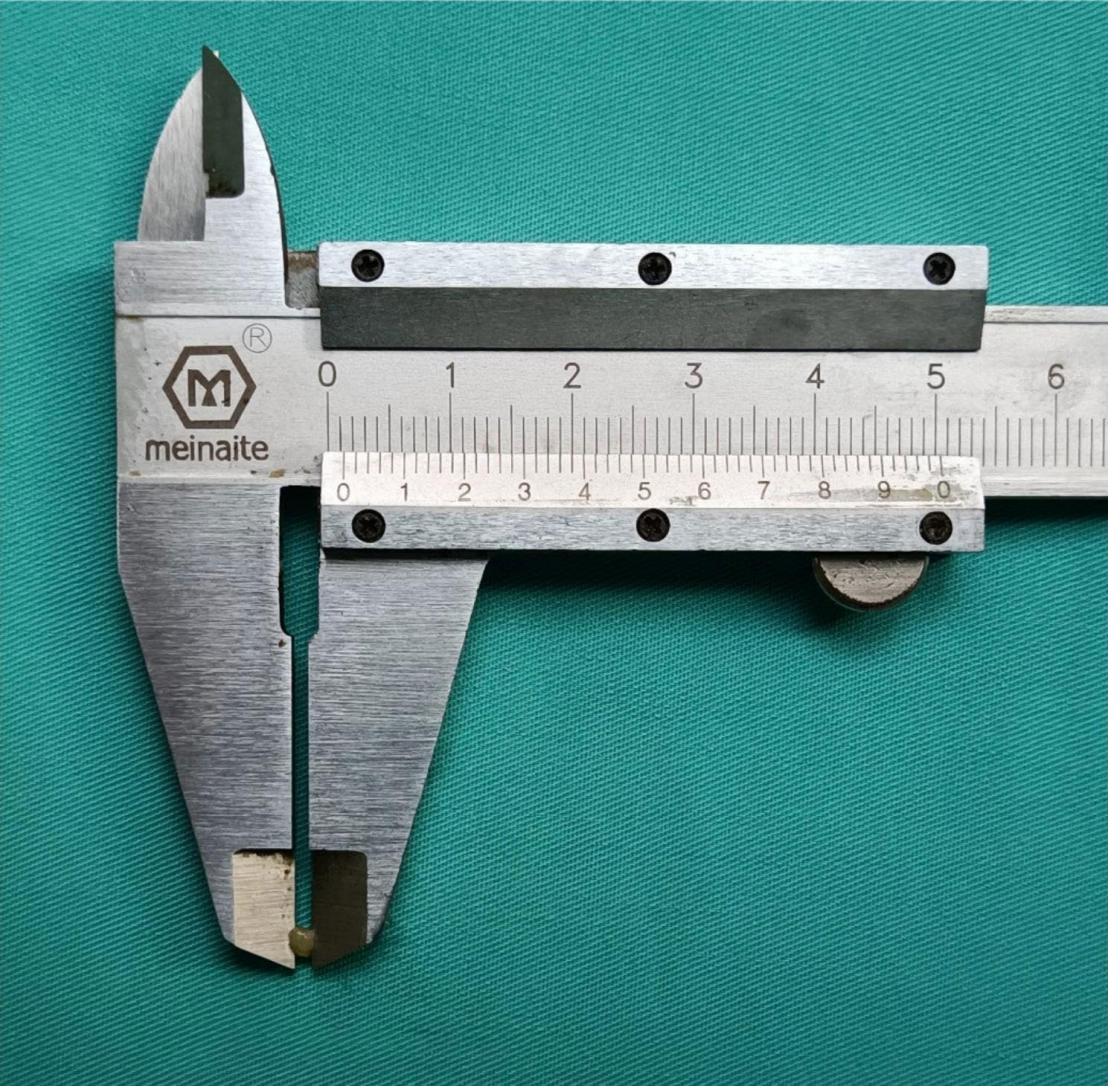
Measuring lymph node diameter with a Vernier calliper

### Surgery

Gastrectomy with D2 lymphadenectomy was performed according to the Japanese classification of gastric carcinoma by the Japanese Gastric Cancer Association [10]. Distal gastrectomy for lymphadenectomy included stations 1, 3, 4sb, 4d, 5, 6, 7, 8a, 9, 11p, and 12a. Proximal gastrectomy for lymphadenectomy included stations 1, 2, 3, 4sa, 4sb, 7, 8a, 9, 10, and 11. Total gastrectomy for lymphadenectomy included stations 1–7, 8a, 9, 10, 11, and 12a. The surgical resection range is determined by the location and extent of the primary tumor. Laparoscopic exploration and detection of free peritoneal cancer cells were the first steps of the surgical procedure to exclude adjacent organ infiltration and peritoneal metastasis. Digestive reconstructions include Billroth II anastomosis after distal gastrectomy and Roux-en-Y anastomosis after total gastrectomy.

### Postoperative retrieval of micro-LNs

After surgery, knowing the location of blood vessels operators should be involved in the retrieval of LNs/micro-LNs. The researchers need to determine the location of LNs in each group, remove excess adipose tissue and preserve the LNs and surrounding soft tissues. Then LNs/micro-LNs are harvested by removing blood vessels, lymphatic vessels, nerve fibers and adipose tissue. After retrieving LNs, researchers measured and recorded the diameter of each LN with a Vernier calliper (Fig. 2).

### Pathological verification

The surgically removed LNs specimens were fixed with 3.7% neutral formaldehyde, paraffin embedded, cut into 2 mm sections, and then stained with hematoxylin and eosin. The LNs were teased as follows. If suspected metastases were visible to the naked eye, representative samples from gross lesions were taken. If no metastases are detected, the largest slice of LNs was selected for pathological analysis. The entire LN was selected for pathological analysis if its maximal transverse diameter of nodes smaller than 5 mm. AE1/AE3-immunostaining was performed to identify carcinoma cells. Positive expression was defined as cells presenting brownish yellow granules located in the cytoplasm. The pathological diagnosis was confirmed by two experienced pathologists with more than 15 years of experience.

### Follow-up

Follow-up was performed every three months in the first year, then every six months for two to five years and every year after five years. The main follow-up included routine physical examination, tumor marker detection (CEA, CA199, CA724 and AFP) abdominal CT, and annual electronic gastroscopy by outpatient follow-up, telephone follow-up and a short message platform follow-up. The overall survival (OS) time was defined as the time between surgery and the last follow-up time or date of death.

### Statistical analysis

SPSS 27.0 statistical software was used for statistical analysis. Count data were analyzed by the chi-square test or Fisher’s exact test. Logistic regression analysis was used to analyze the risk factors for micro-LNM. Survival estimates were calculated using Kaplan‒Meier analyze and a Kaplan‒Meier curve was generated. Univariate and multivariate Cox proportional hazards regression models were used to determine independent prognostic factors for gastric cancer patients, and the hazard ratio (HR) and its 95% CI were estimated. Variables that showed significant results from the univariate analysis were included in the multivariate analysis with backward elimination. *P* < 0.05 indicated statistically significant differences.

## Results

### Patient characteristics

A total of 107 patients (56.02%, 107/191) had LNM, including 85 patients (44.50%, 85/191) with micro-LNM and the incidence of micro-LNM was relatively common in GC patients.

**Fig. 3:**
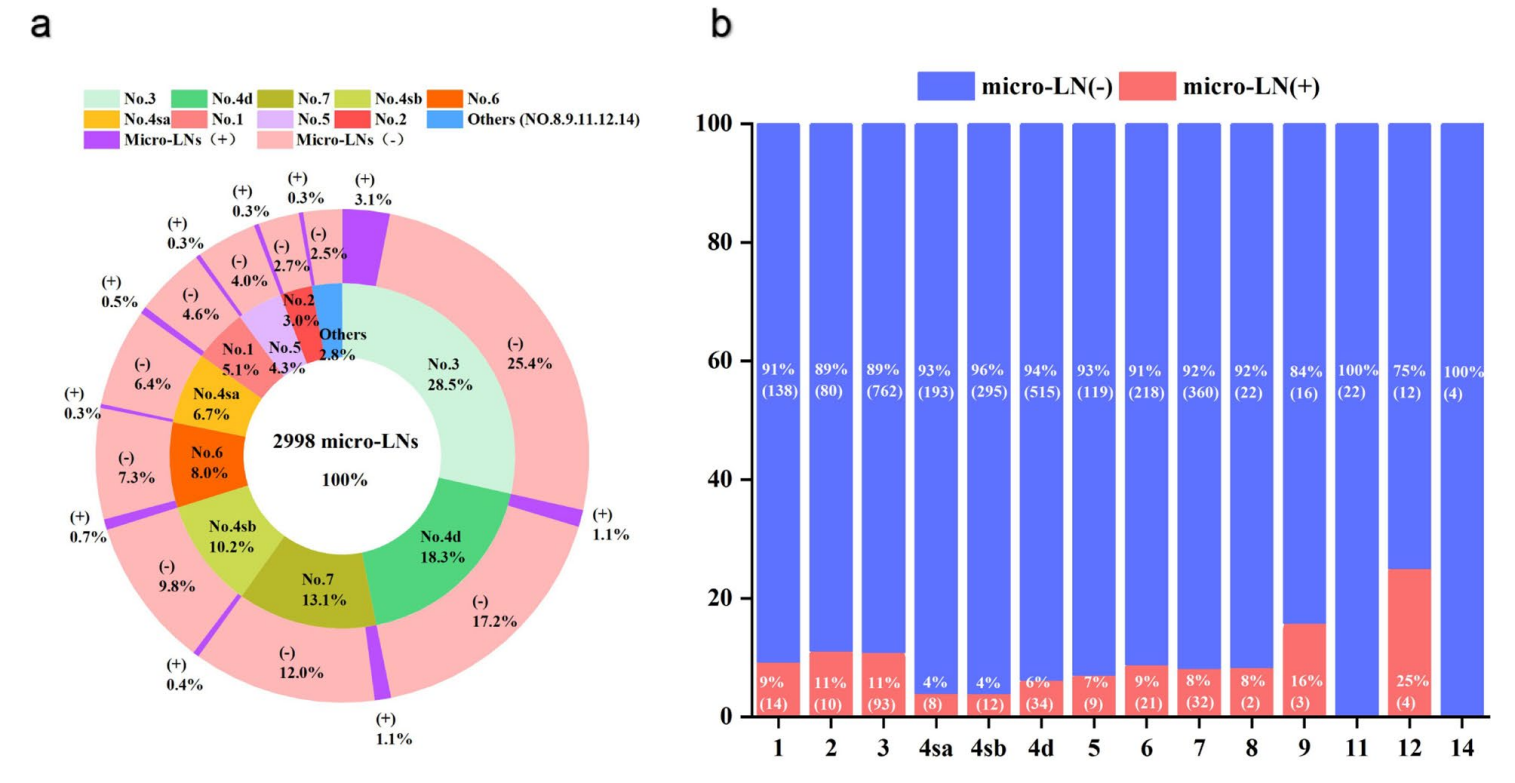
Retrieved micro lymph nodes according to lymph node station. (**a**). The chart depicts the relative distribution of the micro lymph nodes. The internal ring demonstrates the ratio of micro lymph nodes among the stations and the external ring shows the status of micro lymph nodes. (**b**). The bar plot represents the metastatic rate of micro lymph nodes

The total number of LNs in all 191 patients was 10,954, with a mean of 57.35 LNs retrieved from each specimen (range 16–167). The total number of micro-LNs retrieved was 2998 (15.70 LNs per patient, range 2–62), of which 242 (8.10%) were metastases (2.85 LNs per patient, range 0–16). The rate of micro-LNM was 8.10%, which was lower than the rate of LNM (12.63%, 1383/10,954). A total of 2998 micro-LNs were analyzed, and the numbers of micro-LNs at each station were summarized in Fig. 3(a).

As shown in Fig. 3(b), the metastatic rate of micro-LNs at each station from high to low was station 12 (4/16, 25.00%), station 9 (3/19, 15.79%), station 2(10/90, 11.11%), station 3(93/855, 10.88%), station 1(14/152, 9.21%), station 6(21/239, 8.79%), station 8 (2/24, 8.33%), station 7 (32/392, 8.16%), station 5 (9/128, 7.03%), station 4d (34/549, 6.19%), station 4sa (8/201, 3.98%), and station 4sb (12/307, 3.91%). Station 11 and station 14 had no micro-LNMs, and there were no micro-LNs retrieved at station 10 and station 13.

### Risk factors for micro-LNM

**Fig. 4:**
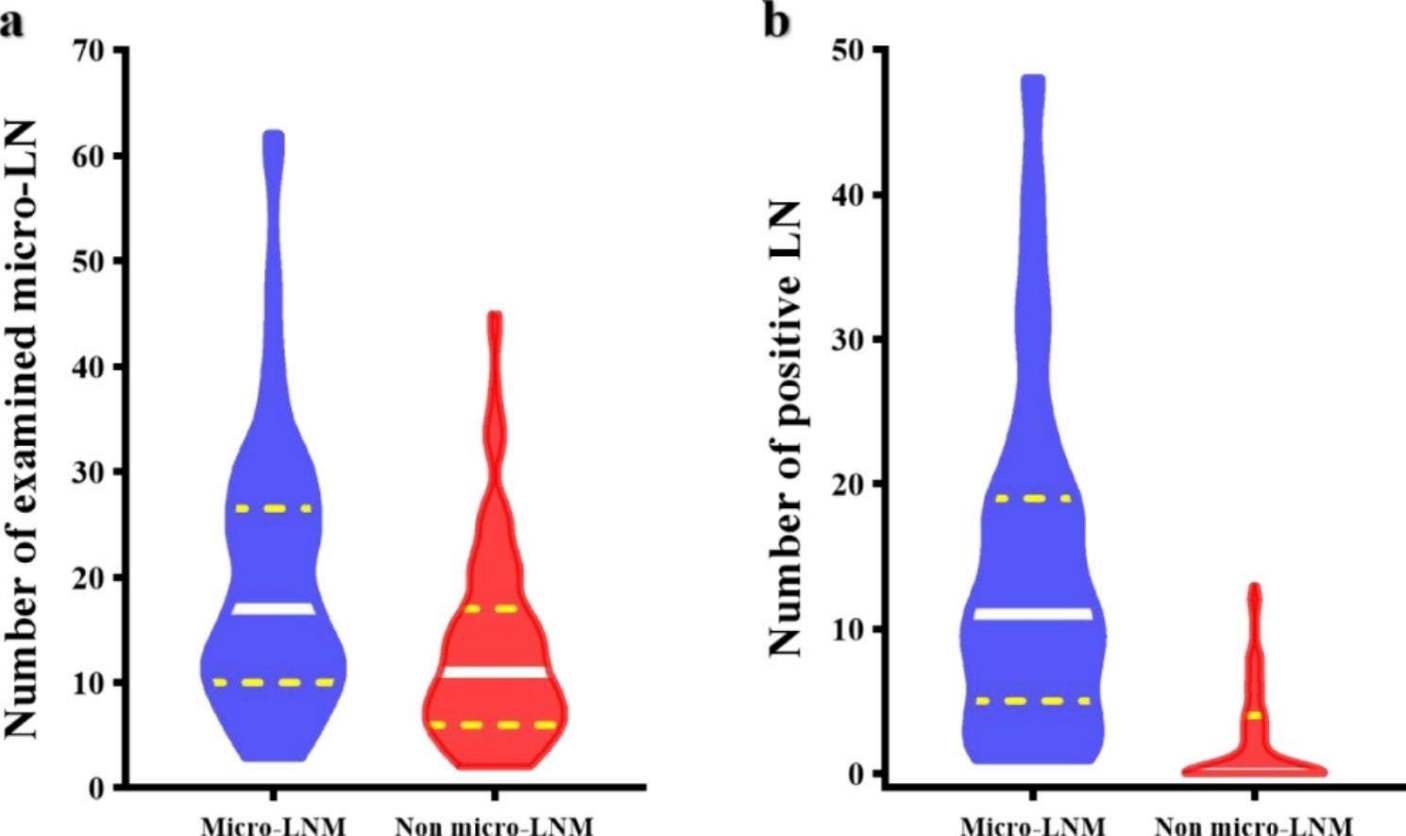
Violin plots of the distribution of the number of lymph nodes. (**a**) shows the distribution of the number of examined micro lymph nodes in patients with and without micro lymph node metastasis. (**b**) shows the distribution of the number of positive lymph nodes in patients with and without micro lymph node metastasis

The clinicopathologic characteristics of GC patients with and without micro-LNM were summarized in Table 1. The results indicated that the patients with micro-LNM had a larger tumor maximum diameter (> 2 cm vs. *≤* 2 cm, 96.5% vs. 64.2%, *P* = 0.000), larger number of examined micro-LNs (*P* < 0.01) (Fig. 4a), larger number of positive LNs (*P* < 0.01) (Fig. 4b), higher levels of CA19-9 (> 30.0U/mL vs. *≤* 30.0U/mL, 35.3% vs. 13.2%, *P* = 0.000) as well as CA72-4 (> 6.9U/mL vs. *≤* 6.9U/mL, 28.2% vs. 9.4%, *P* = 0.001), higher proportion of undifferentiated type (90.6% vs. 56.6%, *P* = 0.000), diffuse type (44.7% vs. 26.4%, *P* = 0.008) and total gastrectomy (45.9% vs. 36.8%, *P* = 0.017), more frequent nerve invasion (57.6% vs. 37.7%, *P* = 0.006) and vascular tumor embolus (30.6% vs. 14.2%, *P* = 0.006), more advanced pathological T category (*P* = 0.018) as well as pathological N category (*P* < 0.001), and greater possibility that cN is inconsistent with pN (81.2% vs. 65.1%, *P* = 0.014). However, the distributions of other clinicopathologic factors including age, gender, the number of retrieved LNs, tumor location, BMI and preoperative CEA and AFP levels were comparable between micro-LN-positive and micro-LN-negative patients. In a multivariate analysis, undifferentiated carcinoma (*P* = 0.034. HR, 3.584; 95%CI, 1.103–11.649), and more advanced pathological N category (*P* < 0.001. N1: HR, 39.632; 95% CI, 4.333–362.473. N2: HR, 50.509; 95% CI, 5.278–483.361. N3: HR, 232.786; 95% CI, 24.243–2235.245) were independent risk factors for micro-LNM. (Table 2).

**Table 1:** Univariate analyses of associations with micro-LNM in patients with GC

Characteristic	All patients	Micro-LN examined		*P* value
		Micro-LNM (N = 85)	Non micro-LNM (N = 106)	
Age (yr)				0.684
≤ 60, N(%)	89 (46.6%)	41(46.1%) 44(43.1%)	48 (45.3%)	
>60, N(%)	102 (53.4%)		58 (54.7%)	
Gender				0.524
Male	137 (71.7%)	59 (69.4%)	78 (73.6%)	
Female	54 (28.3%)	26 (30.6%)	28 (26.4%)	
tumor maximum diameter(cm)				0.000
≤ 2	41 (21.5%)	3(3.5%)	38 (35.8%)	
>2	150 (78.5%)	82(96.5%)	68 (64.2%)	
Number of examined LN, median (25%-quantile, and 75%-quantile)	53 (41;72)	56 (42; 78)	51 (39; 66.25)	0.129
Number of positive LN, median (25%-quantile, and 75%-quantile)	4 (0;11)	11 (5; 19)	0 (0; 4)	< 0.01
Number of examined micro-LN, median (25%-quantile, and 75%-quantile)	13 (8;22)	17 (10; 26.5)	11 (6; 17)	< 0.01
Tumor location				0.478
upper	58 (30.4%)	22 (25.9%)	36 (34.0%)	
middle	43 (22.5%)	20 (23.5%)	23 (21.7%)	
lower	90 (47.1%)	43 (50.6%)	47 (44.3%)	
Type of resection				0.017
Proximal gastrectomy	20 (10.5%)	3 (3.5%)	17 (16.0%)	
Distal subtotal gastrectomy	93 (48.7%)	43 (50.6%)	50 (47.2%)	
Total gastrectomy	78 (40.8%)	39 (45.9%)	39 (36.8%)	
Pathological T category				0.018
T1	53 (27.7%)	14 (16.5%)	39 (36.8%)	
T2	24 (12.6%)	11 (12.9%)	13 (12.3%)	
T3	10 (5.2%)	5 (5.9%)	5 (4.7%)	
T4	104 (54.5%)	55 (64.7%)	49 (46.2%)	
Pathological N category				< 0.001
N0	60 (31.4%)	0	59 (55.7%)	
N0	27 (14.1%)	14 (16.5%)	14 (13.2%)	
N2	33 (17.3%)	15 (17.6%)	18 (16.9%)	
N3	71 (37.2%)	56 (65.9%)	15 (14.2%)	
Histologic type				0.000
Differentiated	54 (28.3%)	8 (9.4%)	46 (43.4%)	
Undifferentiated	137 (71.7%)	77 (90.6%)	60 (56.6%)	
Nerve invasion				0.006
Yes	89 (46.6%)	49 (57.6%)	40 (37.7%)	
No	102 (53.4%)	36 (42.4%)	66 (62.3%)	
Vascular tumor embolus				0.006
Yes	41 (21.5%)	26 (30.6%)	15 (14.2%)	
No	150 (78.5%)	59 (69.4%)	91 (85.8%)	
Lauren classification				0.008
Diffuse	66 (34.6%)	38 (44.7%)	28 (26.4%)	
Intestinal and Mixed	125 (65.4%)	47 (55.3%)	78 (73.6%)	
BMI(kg/m^2^)				0.609
< 18.5	12 (6.3%)	7 (8.2%)	5 (4.7%)	
18.5–23.9	76 (39.8%)	33 (38.8%)	43 (40.6%)	
> 23.9	103 (53.9%)	45 (53.0%)	58 (54.7%)	
CEA(ng-/mL)				0.083
≤ 5.0	142 (74.3%)	58 (68.2%)	84 (79.2%)	
> 5.0	49 (25.7%)	27 (31.8%)	22 (20.8%)	
AFP(ng/mL)				0.302
≤ 7.0	178 (93.2%)	81 (95.3%) 4 (4.7%)	97 (91.5%)	
> 7.0	13 (6.8%)		9 (8.5%)	
CA19-9(U/mL)				0.000
≤ 30.0	147 (77.0%)	55 (64.7%)	92 (86.8%)	
> 30.0	44 (23.0%)	30 (35.3%)	14 (13.2%)	
CA72-4(U/mL)				0.001
≤ 6.9	157 (82.2%)	61 (71.8%)	96 (90.6%)	
>6.9	34 (17.8%)	24 (28.2%)	10 (9.4%)	
cN is consistent with pN				0.014
Yes	53 (27.7%)	16(18.8%)	37 (34.9%)	
No	138(75.3%)	69(81.2%)	69 (65.1%)	

**Table 2:** Multivariate logistic regression analyses for micro-LNM

Characteristic	Odds ratio	95%CI	P value
Tumor maximum diameter (cm, > 2 vs. ≤ 2)	5.945	0.914–38.671	0.062
Histologic type (Undifferentiated vs. Differentiated)	3.584	1.103–11.649	0.034
Nerve invasion (yes vs. no)	0.603	0.231–1.5786	0.303
Vascular tumor embolus (yes vs. no)	1.075	0.412–2.803	0.883
Lauren classification (Diffuse vs. Intestinal and Mixed)	0.938	0.374–2.357	0.892
*Pathological T category* (ref. T1)			0.226
T2	0.836	0.131–5.320	0.850
T3	0.526	0.053–5.206	0.583
T4	0.265	0.053–1.337	0.108
*Pathological N category* (ref. N0)			< 0.001
N1	39.632	4.333–362.473	0.001
N2	50.509	5.278–483.361	< 0.001
N3	232.786	24.243–2235.245	< 0.001

### Survival analysis

**Fig. 5:**
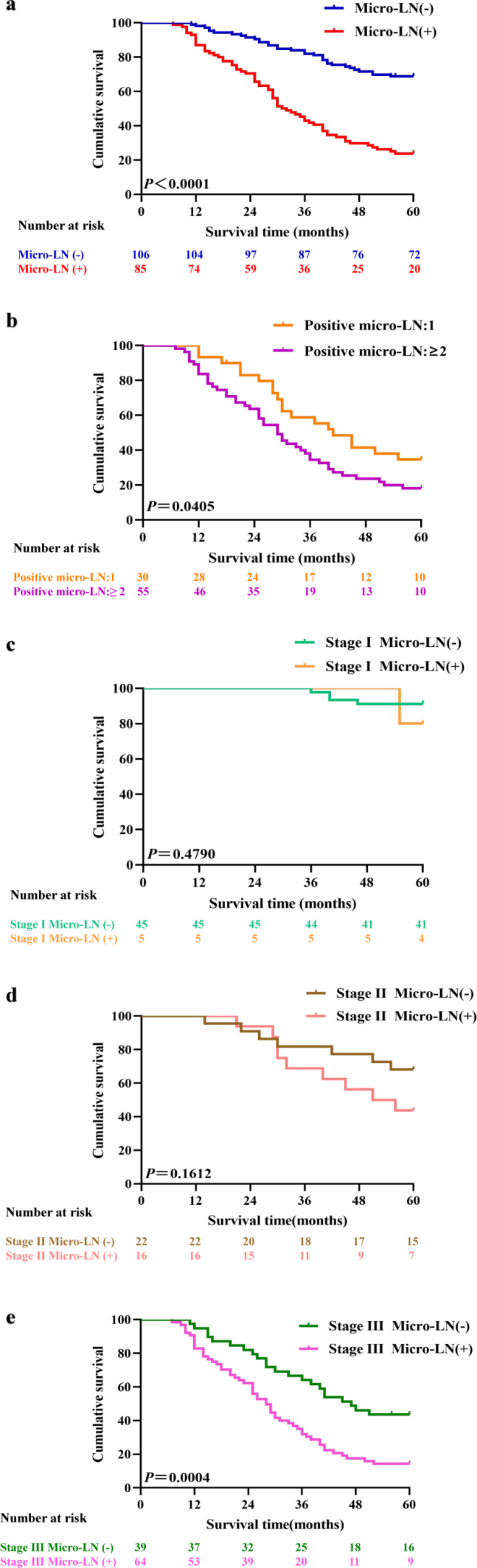
Kaplan‒Meier curve of overall survival. (**a**). Kaplan‒Meier estimate of all 191 enrolled patients by micro lymph node positivity. (**b**). Kaplan‒Meier estimate of overall survival by the number of positive micro lymph nodes. (**c**). Kaplan‒Meier estimate of stage I patients by micro lymph node positivity. (**d**). Kaplan‒Meier estimate of stage II patients by micro lymph node positivity. (**e**). Kaplan‒Meier estimate of stage III patients by micro lymph node positivity

The median follow-up duration was 55.0 months, and the 5-year OS rate for all 191 enrolled patients was 49.2%. The Kaplan–Meier survival curve (Fig. 5a.) showed that patients with micro-LNM experienced poorer survival than micro-LN-negative patients (5-year OS: 24.7% vs. 68.9%; *P* < 0.0001). The survival outcome of the patients with two or more positive micro-LNs was worse than that of those with 1 positive micro-LN (5-year OS: 18.2% vs. 36.7%, *P* < 0.05). Kaplan–Meier curves for OS based on the numbers of positive micro-LNs are shown in Fig. 5b. Subgroup analysis was also performed. The 5-year OS rates of stage I patients with and without micro-LNM were 80.0% and 91.1%, respectively; there was no significant survival difference between the two groups (*P* = 0.4790). The similar finding was observed in stage II patients (5-year OS rate, micro-LN-positive 43.8% vs. micro-LN-negative patients 68.2%, *P* = 0.1612). For stage III patients, the 5-year OS rates of patients with and without micro-LNM were 15.6% and 43.6%, respectively (*P* = 0.0004). Based on the above results, the prognostic impact of micro-LNM in stage III patients was determined. Kaplan–Meier curves for OS based on stage are shown in Fig. 5c-e.

Table 3 shows the results of Cox univariate and multivariate analysis. According to the univariate Cox regression analyses, micro-LNM, age, histologic type, nerve invasion, vascular tumor embolus, tumor maximum diameter, pathological T category and N category were associated with the OS of GC patients (all *P* < 0.05). The multivariate analysis demonstrated that micro-LNM (HR:2.199, 95% CI 1.335–3.622, *P* = 0.002), pathological T category (T2 category, HR:3.345, 95% CI 1.208–9.262, *P* = 0.020; T3 category, HR:3.991, 95% CI 1.152–13.821, *P* = 0.029; T4 category, HR:4.807, 95% CI 1.985–11.640, *P* < 0.001) as well as pathological N category (N1 category, HR:2.349, 95% CI 0.855–6.450, *P* = 0.098; N2 category, HR:3.390, 95% CI 1.277–8.998, *P* = 0.014; N3 category, HR:4.130, 95% CI 1.575–10.834, *P* = 0.004) were independent prognostic factors for OS in gastric cancer patients.

**Table 3:** Univariate and multivariate Cox analysis of postoperative survival time

Characteristic	Univariate analysis			Multivariate analysis	
	HR (95%CI)	*P* value		HR (95%CI)	*P* value
Micro-LNM (positive vs. negative)	3.833(2.508–5.859)	< 0.001		2.199(1.335–3.622)	0.002
Age (> 60years vs. ≤ 60 years)	1.575(1.045–2.374)	0.030		1.391(0.888–2.180)	0.149
Histologic type(Differentiated vs. Undifferentiated)	2.639(1.542–4.515)	< 0.001		1.062(0.571–1.973)	0.849
Nerve invasion (yes vs. no)	2.963(1.953–4.495)	< 0.001		1.441(0.894–2.322)	0.133
Vascular tumor embolus (yes vs. no)	2.012(1.300–3.113)	0.002		1.050(0.654–1.687)	0.839
Tumor maximum diameter (> 2 cm vs. ≤ 2 cm)	4.387(2.126–9.055)	< 0.001		0.821(0.350–1.927)	0.650
Pathological T category (ref. T1)		< 0.001			0.006
T2	4.891(1.923–12.441)	< 0.001		3.345(1.208–9.262)	0.020
T3	5.310(1.684–16.746)	0.004		3.991(1.152–13.821)	0.029
T4	9.061(4.160–19.734)	< 0.001		4.807(1.985–11.640)	<0.001
Pathological N category (ref. N0)		< 0.001			0.028
N1	4.937(1.968–12.382)	< 0.001		2.349(0.855–6.450)	0.098
N2	8.916(3.784–21.006)	< 0.001		3.390(1.277–8.998)	0.014
N3	12.623(5.729–27.812)	< 0.001		4.130(1.575–10.834)	0.004
Lauren classification (Intestinal and Mixed vs. Diffuse)	0.759(0.504–1.144)	0.187		1.029(0.636–1.665)	0.907

## Discussion

LNM is the most common metastatic pattern and the crucial prognostic factor in patients with GC [11]. D2 lymphadenectomy has gradually become the standard surgery in advanced gastric cancer [12, 13] and includes the dissection of micro-LNs. Current GC staging is based on the TNM system and N category is based solely on the number of metastatic LNs. A more advanced pathological N category indicates a more severe condition and predicts a poor prognosis. However, some GC patients with the same TNM stage who underwent the same treatment regimen have different clinical outcomes indicating that these patients may obtain inaccurate staging causing inaccurate assessments of prognosis. In this paper, we focused on the influence of micro-LNs on prognosis and stage migration. To the authors’ knowledge, none have specifically studied the relations between micro-LNM and prognosis in GC.

Our study found that micro-LNM was an independent prognostic factor and was of common occurrence in GC patients. In a Japanese study, the authors measured the diameters of 3124 positive LNs and found 37.8% were less than 5 mm [14]. Mönig et al. reported that metastatic LNs less than 3 mm in diameter accounted for 14.5% of all metastatic nodes [15]. This research also found the diameter of approximately 20.5% of examined LNs was less than 2 mm according to the results of a previous study [8]. In our study, a LN maximum diameter *≤* 2 mm was used as a parameter to define micro-LN. Our study found that there were micro-LNM in LNs No. 1, 2, 3, 4sa, 4sb, 4d, 5, 6, 7, 8, 9, and 12 in clinical practice, and the rate of micro-LNM in LN No. 12 was 25.0%. Although part of the reason for the high micro-LN metastasis rate in LN No. 12 is that our number of harvested micro-LNs is relatively small, the existence of positive micro-LNs indicates D2 lymphadenectomy is worth doing in GC surgical management. Micro-LNs are included in D2 LN dissection. Surgeons may need to place great importance on micro-LNs and dissect the perigastric LNs completely and thoroughly. The practice of relying on vision and palpation to identify nodal metastasis is rated undesirable and unsafe [16]. The results of this study showed that micro-LNM could be regarded as a supplementary predictor for survival in GC patients. Large sample, multicenter, randomized clinical trials are still needed in the future. The present research does not think metastases in micro-LNs are more important than metastases in larger LNs, but micro-LNM has unique implications and deserves clinical attention.

Sufficient retrieval of LNs from the specimen is critical for LN staging [17, 18]. A minimum of 16 LNs should be retrieved according to the 8th edition of the AJCC staging manual [12, 19]. Some researchers thought that it was not adequate to retrieve at least 16 LNs to obtain an accurate pathological stage and suggested that at least 30 LNs should be retrieved in stage II patients [20]. Increasing the number of LNs retrieved implies not only a relatively more thorough LN dissection but also a more accurate N category. After the analysis of clinicopathological data and survival results of 2455 GC patients, Deng et al. found that increasing the number of examined LNs is a prerequisite to guaranteeing precise TNM classification [21]. Currently, the assessment of GC prognosis relies on accurate TNM staging. Retrieval of micro-LNs will be an effective way to increase the number of LNs retrieved. If all micro-LNs are ignored in our study, approximately 27.4% of LNs and 17.5% of positive LNs will be missed that can cause severe stage migration or false pN staging. The retrieval of LNs, especially micro-LNs, that should be completed by surgeons cannot be separated from the support of the pathology department [22]. When LNs were transferred to the pathology department, the LNs identifiers (station, size, number, etc.) should be recorded clearly.

The finding of positive micro-LNs has put forward higher requirements for imaging technology. Currently, the clinical stage of GC primarily relies on CT, but the LN status evaluated by CT is usually highly inaccurate [23–25]. The primary reason for this inaccuracy is that the preoperative assessment of LN status is mainly based on the size of LNs. A LN is often suspected to be positive if its short axis diameter > 10 mm [3, 26, 27]. Our study found metastasis in LNs could exist regardless of the size. Among the 85 patients with micro-LNM, clinical N category was consistent with pathological staging in 16 cases (18.8%), but 34.9% (37/106) of patients without micro-LNM obtained the same N category. The difference had statistical significance. The preoperative assessment of GC patients cannot rely only on clinical TNM staging. Surgeons should make a comprehensive assessment with all the available pathological features. Patients prior to surgery with tumor maximum diameter > 2 cm, CA19-9 > 30.0 U/ml, CA72-4 > 6.9 U/mL, and more advanced clinical N category are more likely to develop micro-LNM based on our current study. Surgeons need to pay sufficient attention to such patients, formulate comprehensive diagnosis and treatment plans and dissect micro-LNs intraoperatively and postoperatively. Micro-LNM indicates poor prognosis and patients with more than two positive micro-LNs had a poorer prognosis than patients with one metastatic micro-LN (*P* < 0.05). In addition to effective and safe adjuvant chemotherapy, the follow-up of patients with micro-LNM needs to be intensified.

The detection of micro-LNs helps to standardize surgical procedure. That micro-LNs are missed often means incomplete LN dissection. Inadequate retrieval of micro-LNs may lead to inaccurate staging migration and assessment of patient condition. There are some challenges and difficulties of the work of retrieving of micro-LNs. Surgeons are required to know the intraoperative situation and the anatomical location information of LNs in the ex vivo specimen, so as to sort out the micro-LNs. Further, the clinical applications of lymphatic tracer, especially carbon nanoparticles suspension injection and indocyanine green, that makes LNs more readily detected have significantly improved the detection number of micro-LNs [6, 8].

Neoadjuvant chemotherapy has become the standard of care in Eastern and Western countries and has been increasingly used in the treatment of advanced GC patients [28, 29]. Neoadjuvant chemotherapy has the advantages of increasing the possibility of a successful R0 resection, eradicating the potential LNM and reducing mortality [30]. Several studies have found that areas of fibrosis and hyalinosis could be found in the positive LNs of GC or breast cancer patients who had undergone neoadjuvant chemotherapy [31, 32]. Moreover, a study by Govindarajan A et al. [33] showed neoadjuvant therapy decreased the number of LNs harvested, because a large proportion of LNs destroyed by chemotherapy drugs became difficult to detect. For the moment, the impact on the micro lymph nodal status of neoadjuvant chemotherapy remains unclear. There were limitations to this study. Firstly, this study was a single-center retrospective study with a possible sample bias and small sample size. Multicenter studies with a larger sample size are needed. Secondly, patients who received neoadjuvant chemotherapy were not included. The impact of neoadjuvant therapy on micro-LNs is unknown. Thirdly, research on the micro-LNs of patients with unresectable gastric cancer is difficult to complete.

## Conclusions

This study reported that the retrieval of micro-LNs helped surgeons perform accurate staging and improve individualized treatment and micro-LNM could be a predictor of survival outcomes in GC patients. Micro-LNs, as a special type of LNs, are not easy to retrieve, but prognosis is poor once metastasis occurs. Patients with risk factors for micro-LNM should arouse the attention of surgeons.

## Data Availability

The participant data with identifiers used to support the findings of this study were supplied by Qun Zhao under license and so cannot be made freely available. Requests for access to these data should be made to Qun Zhao, zhaoqun@hebmu.edu.cn.

## Abbreviations

GC: gastric cancer
LNM: lymph node metastasis
LN: lymph node
CT: computed tomography
micro-LN: micro lymph node
micro-LNM: micro lymph node metastasis
LNMM: lymph node micrometastasis
OS: overall survival
HR: hazard ratio.
